# Supplementary figures and images for: Pyroptosis-Related Gene Signatures Can Robustly Diagnose Skin Cutaneous Melanoma and Predict the Prognosis
Source: Front Oncol. 2021 Jul 13;11:709077. doi: 10.3389/fonc.2021.709077 (PMC8313829; doi:10.3389/fonc.2021.709077)

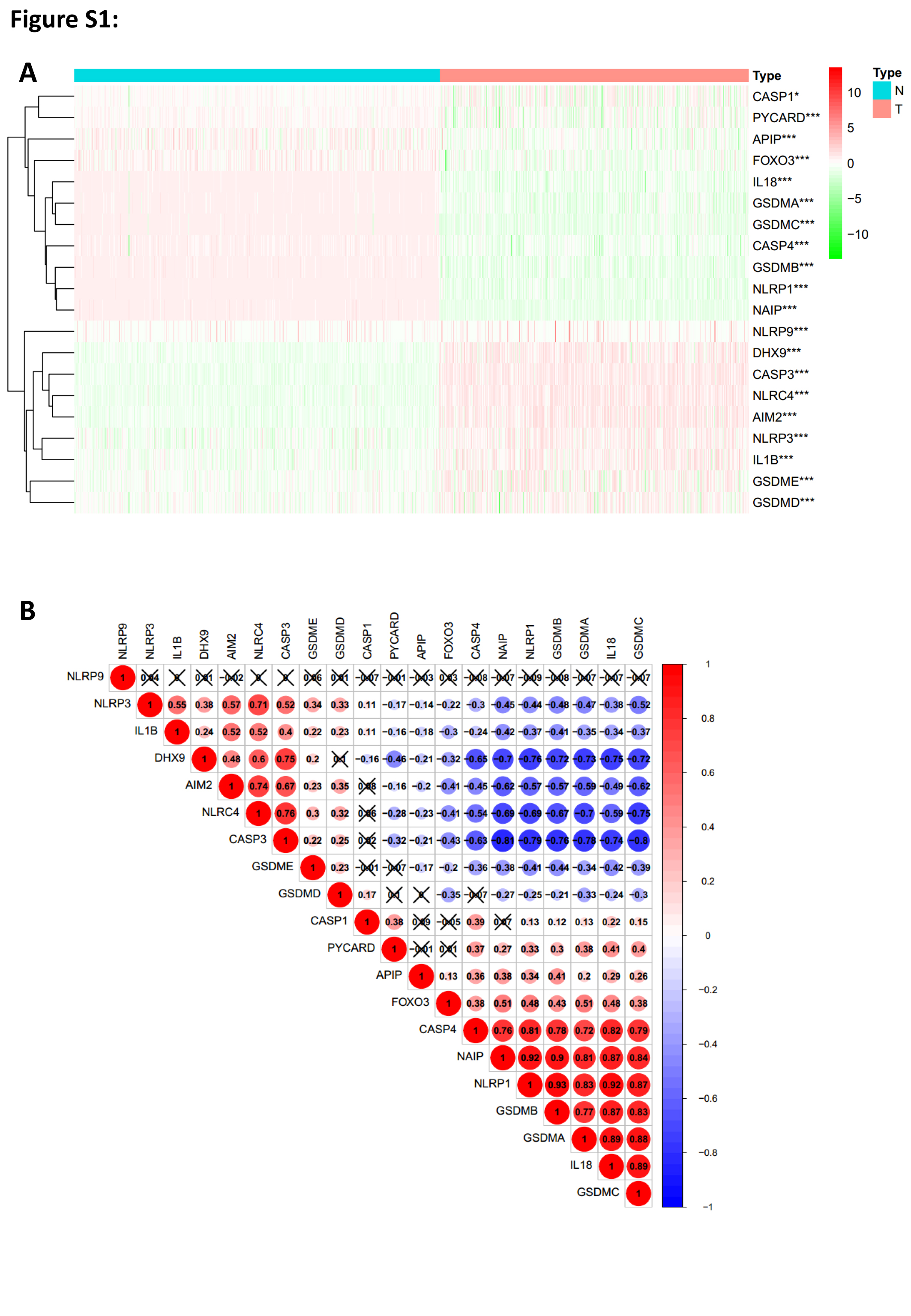

Supplement: Supplementary Figure 1 — Differentially expressed PRGs and the correlations in expression. (A) Heatmap of differentially expressed PRGs in TCGA-SKCM & GTEx-SKIN (*p < 0.05, **p < 0.01, ***p < 0.001). (B) Bubble graph for PRGs (the bigger bubble and the increasing depth of red means higher significance). [file Image_1.jpeg]

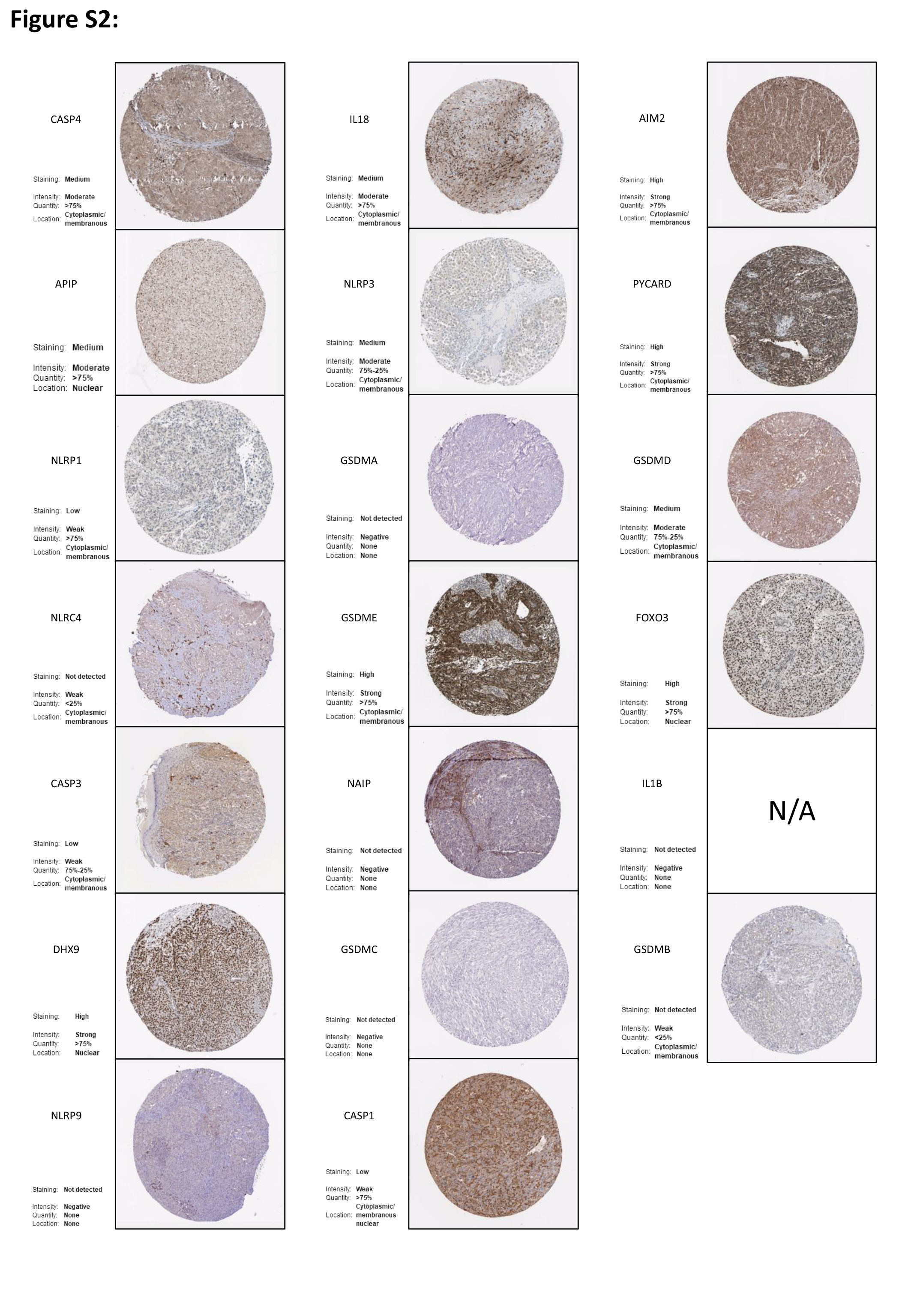

Supplement: Supplementary Figure 2 — Protein levels of PRGs in SKCM. Immunohistochemistry staining images of proteins encoded by PRGs in SKCM were retrieved from the Human Protein Atlas (www.proteinatlas.org). [file Image_2.jpeg]

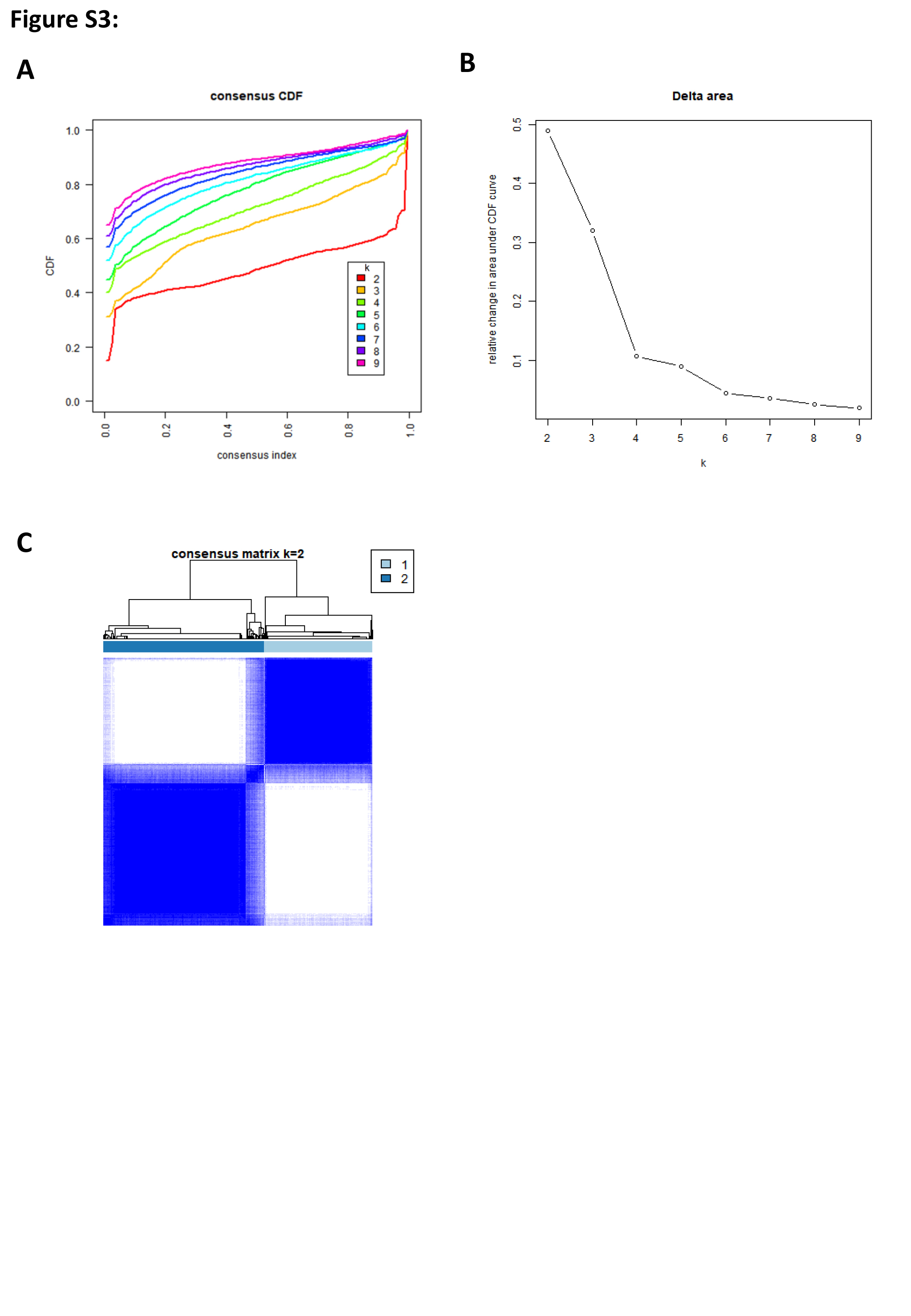

Supplement: Supplementary Figure 3 — Tuning of k value for consensus clustering matrix. (A) CDF curves in consensus clustering (B) Relative changes in the AUC of CDF curves. (C) Consensus clustering matrix. [file Image_3.jpeg]

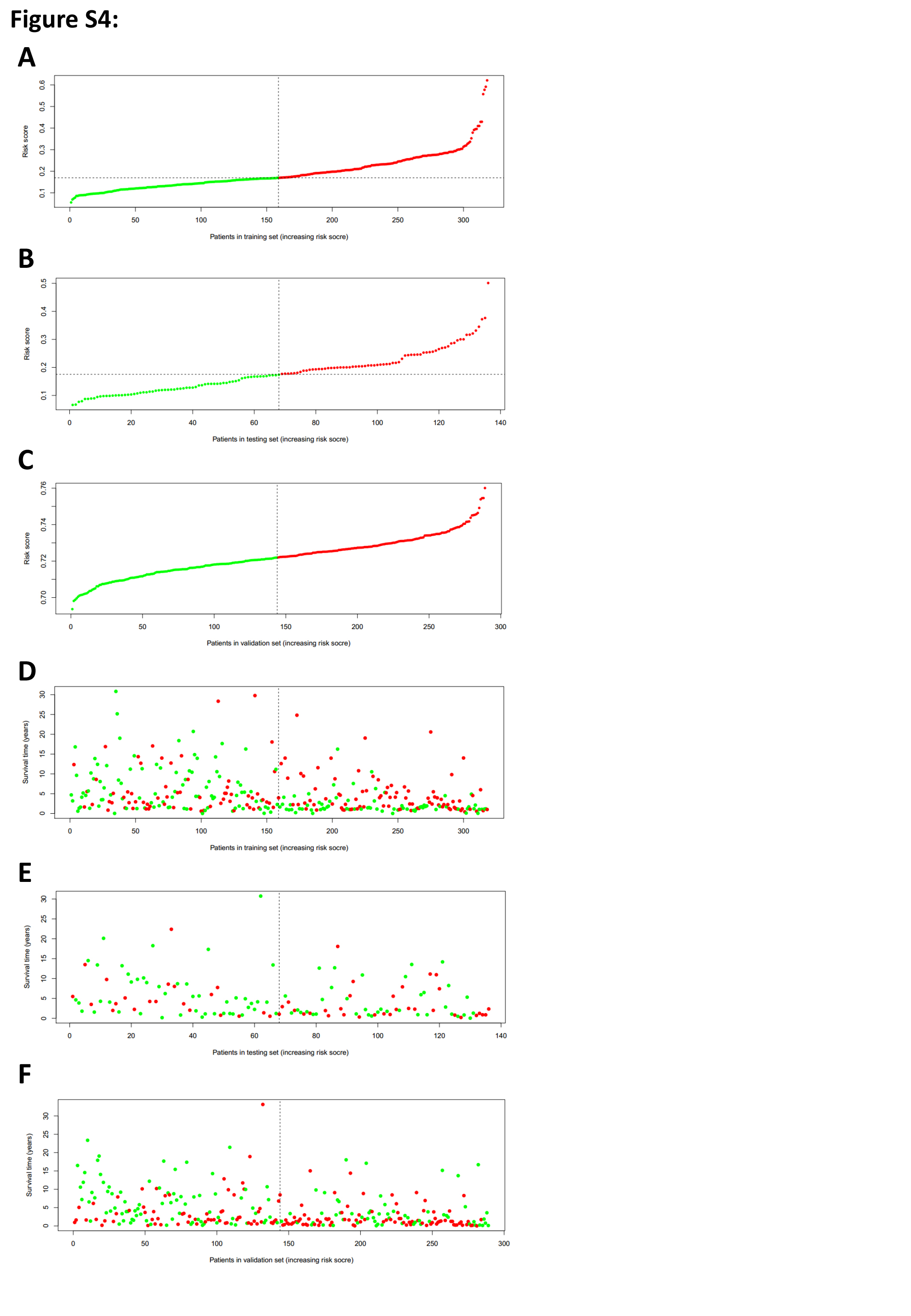

Supplement: Supplementary Figure 4 — Distribution of patients based on the risk score. (A–C) Distribution of patients based on the risk score in training set (A), testing set (B), and validation set (C). (D–F) Distribution of survival time based on the risk score in training set (D), testing set (E), and validation set (F). [file Image_4.jpeg]

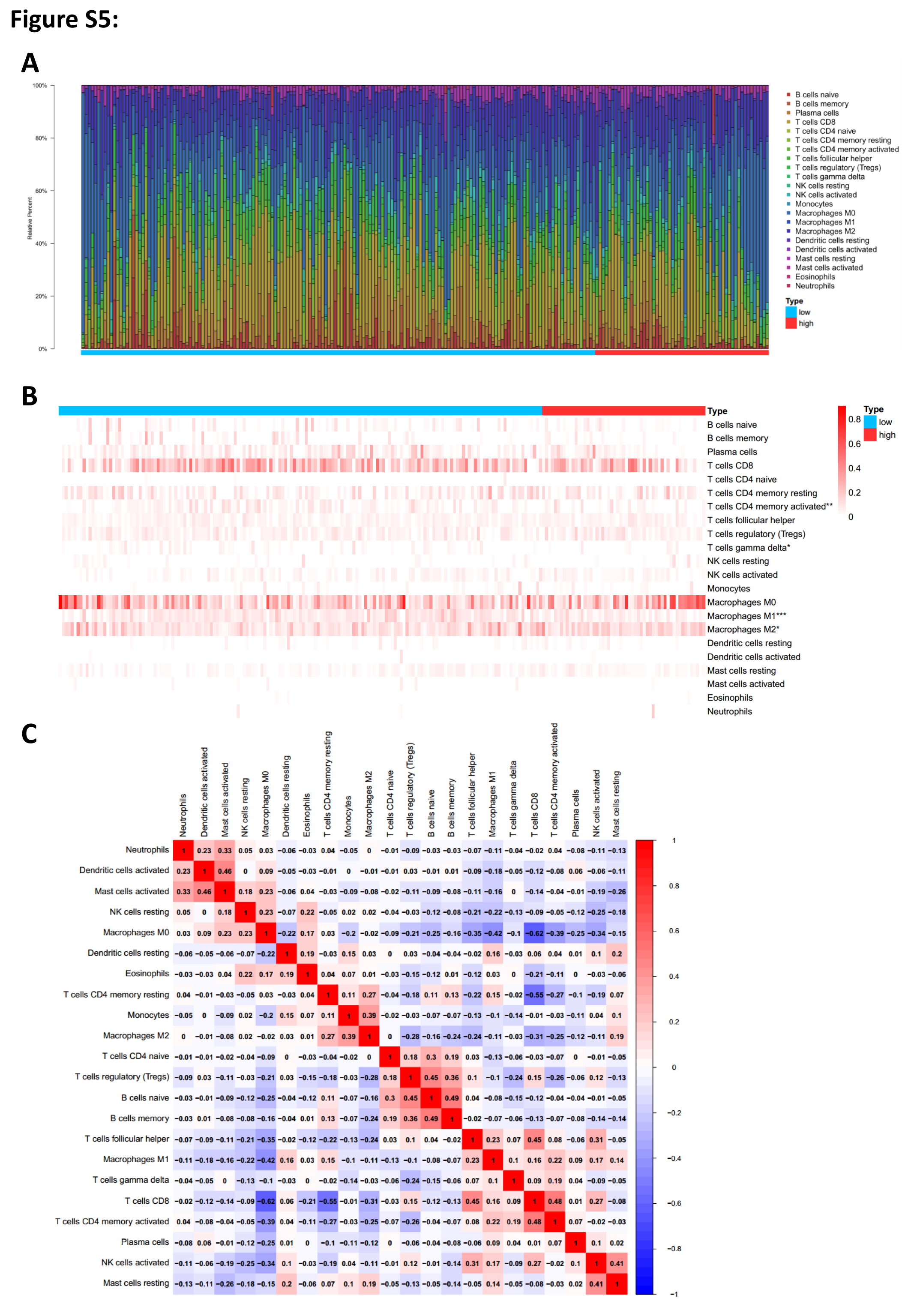

Supplement: Supplementary Figure 5 — Analyses of immune microenvironment. (A) Proportional histogram of the percentage of each immune cell. (B) Heatmap showing the amount of each immune cell in patients of TCGA-SKCM. (C) Heatmap showing the relationships among immune cells (the increasing depth of red means higher significance). [file Image_5.jpeg]

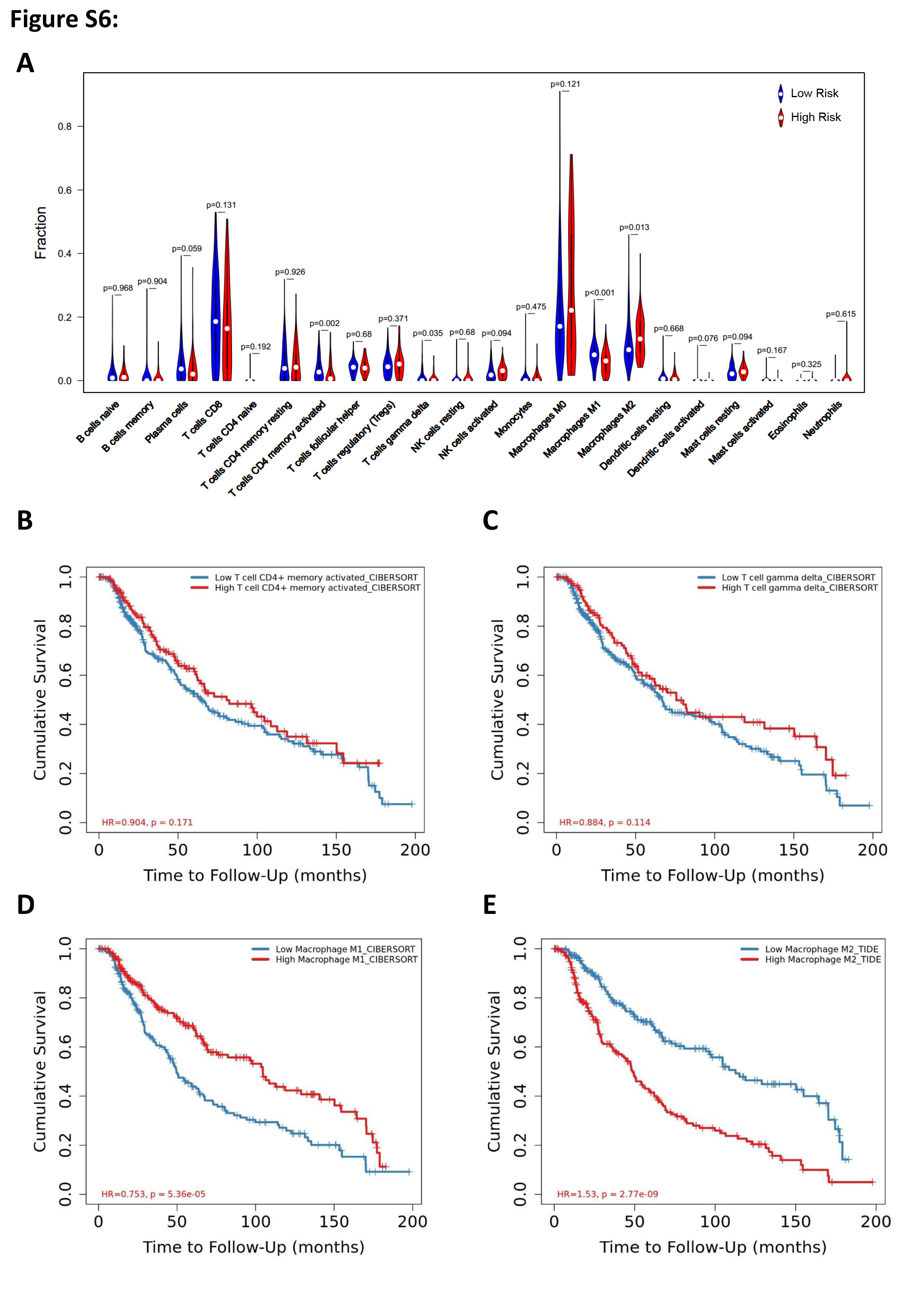

Supplement: Supplementary Figure 6 — Comparison of the immune microenvironment between subgroups. (A) Violin plot showing the relationship between the risk score and immune fractions. The red color represents the high-risk subgroup while the blue color represents the low-risk subgroup. (B–E) KM curves showing the relationship between cumulative survival and immune cells in significantly different infiltrating levels, including activated CD4+ memory T cells (B), γδ T cells (C), M1 macrophages (D), and M2 macrophages (E). HR, hazard ratio. [file Image_6.jpeg]
